# First Evidence of Simultaneous Circulation of Three Different Dengue Virus Serotypes in Africa

**DOI:** 10.1371/journal.pone.0078030

**Published:** 2013-10-21

**Authors:** Melanie Caron, Gilda Grard, Christophe Paupy, Illich Mamfred Mombo, Branly Bikie Bi Nso, Fabrice Roland Kassa Kassa, Dieudonne Nkoghe, Eric Maurice Leroy

**Affiliations:** 1 Centre International de Recherches Médicales de Franceville (CIRMF), Franceville, Gabon; 2 Institut de recherche pour le développement (IRD), Montpellier, France; 3 Ministère de la Santé Publique, Libreville, Gabon; Tulane School of Public Health and Tropical Medicine, United States of America

## Abstract

Gabon, in Central Africa, was affected for the first time in 2007 and then in 2010 by simultaneous outbreaks of chikungunya and Dengue serotype 2 (DENV-2) viruses. Through the national surveillance of dengue-like syndromes between 2007 and 2010, we observed continuous circulation of DENV-2 in a southward movement. This rapid spread of DENV-2 was associated with the emergence of DENV-1 in 2007 and DENV-3 in 2010. Interestingly, we detected six DENV-2 infected patients with hemorrhagic signs during the second outbreak in 2010. Although these cases do not meet all standard WHO criteria for severe Dengue with hemorrhage (formerly DHF), this is the first report of several dengue fever cases associated with hemorrhagic signs during a simultaneous circulation of different DENV serotypes in Africa. Together, these findings suggest that DENV is becoming more widely established on this continent and that DHF will likely become a serious public-health problem in the near future.

## Introduction

Dengue fever (DF) has a major impact on human health around the world, with up to 50 million cases annually in over 100 endemic countries located mainly in the Asia-Pacific and Americas-Caribbean regions [1]. More than one-third of the global population is at risk of potentially life-threatening complications associated with severe Dengue with hemorrhage (formerly DHF) and with shock syndrome (formerly DSS). Typical DF includes fever, headache, arthralgia, myalgia and rash, but Dengue virus (DENV) infections cause a broad spectrum of clinical forms ranging from asymptomatic to severe, the latter being characterized by increased vascular permeability or hypovolemia [2]. Each year about 500,000 DF patients require hospitalization, with a mortality rate of about 2.5%, mainly in childhood [3].

DF is one of the most important emerging mosquito-borne infections worldwide. DENV has a long history of human infection in tropical and subtropical climates. The DENV transmission probably occurs through a sylvatic cycle involving non-human primates and simiophilic *Aedes* mosquito species, and mainly through an urban cycle involving humans and anthropophilic *Aedes* mosquito species [4]. Although *Ae. aegypti* is regarded as the main vector of DENV outbreaks worldwide, *Ae. albopictus* has also been described as a major vector, especially in territories where it has gradually replaced local species such as *Ae. aegypti* [5].

DENV belongs to the *Flaviviridae* family genus Flavivirus, and comprises four viruses (DENV-1 to DENV-4) that are genetically related but antigenically distinguishable. Extensive DENV genetic diversity within the different serotypes, themselves structured into distinct genotypes, has been highlighted by phylogenetic investigations, notably focusing on the envelope (E) protein [6]. This has allowed researchers to trace the geographic origins and expansion of DENV throughout the world. Although views differ, DENV is widely thought to have emerged in Asia. Based on phylogenetic data, the topology of Asian lineages is shown by the deep and consistent location of ancient isolates from which recent isolates arose, defining a viral evolution radiating around a spatial clade [7]. This is also supported by the fact that all four DENV serotypes are present in Asia and cause regular outbreaks. Indeed, the longer DENV circulates in a given area, the more the emerging DENV serotypes, such as in Malaysia where circulation of multiple DENV serotypes is commonly described [6]. Furthermore, circulation of multiple DENV serotypes may lead to concurrent infections. In India for instance, cases of human infection with all four DENV serotypes have even been reported [8].

One aggravating factor in DF is subsequent infection by a different DENV serotype. Initial studies conducted in Thailand showed that DHF/DSS was up to 15-80 times more frequent in patient who had experienced DF and had then been infected by a different DENV serotype [9]. Consequently, DHF/DSS is usually associated with simultaneous circulation of different DENV serotypes, particularly when primary infection with DENV-1 is followed by DENV-2 or DENV-3 [10]. In Brazil, a dramatic DHF/DSS increase has been observed over the last 20 years in conjunction with the emergence of multiple DENV serotypes [11]. Based on both *in vitro* studies and indirect evidences in human, antibody-dependent enhancement (ADE) has been proposed as the main mechanism underlying DHF/DSS: preexisting immunity to a given DENV serotype enhances the severity of a secondary infection by a different DENV serotype [12,13]. In the DENV infection course, formation of antibody-virus complex enhances the intensity of infection and/or the number and/or types of cells infected, thereby increasing viremia and consequent disease severity. Presumably, subneutralizing concentrations of a heterotypic antibody leads to ADE. Indeed, up to 99% of DHF patients were found to have heterotypic antibodies to the DENV serotype responsible for the DHF [14]. By contrast with the observations made in Asia and the Americas [6,8,11], only three cases clinically compatible with a DHF have been reported to date in Africa: several years ago in Mozambique and Djibouti, and recently in Guinea Bissau from a traveler infected with a sylvatic DENV-2 strain [15,16]. Despite the effective DENV circulation such as DF acquired by travelers from African countries [17], outbreaks involving multiple DENV serotypes are rarely observed in Africa and data remain scarce on this continent.

Following the 2007 Gabon outbreak [18,19], we conducted active surveillance of dengue-like syndromes throughout the country. Between 2007 and 2010, we observed rapid and continuous spread of DENV from north-west to south-east Gabon in conjunction with the emergence of multiple DENV serotypes and the appearance of severe clinical forms of DF.

## Methods

### Ethics Statement

Following the 2007 Gabon outbreak [18,19], the public-health response was based on cooperation between the Gabonese Ministry of Health (MoH) and ‘Centre International de Recherches Médicales de Franceville’ (CIRMF). The study was approved by an Institutional Review Board (‘Conseil Scientifique du CIRMF’) and approved by the Regional Health Director. Given the urgency of diagnosis and according to the MoH directives, only individual oral consent was required for blood sampling. Results were transmitted to patients and to the MoH.

### DF and DHF case definitions

In keeping with standard WHO case definitions [20], DF was defined by acute fever (>38.5°C) and at least two of the following symptoms: headache, retro-orbital pain, arthralgia, myalgia, rash, hemorrhagic signs or leucopenia; while DHF was defined as DF combined with spontaneous bleeding, thrombocytopenia and increased vascular permeability (hypoproteinemia or altered hematocrit). Clinical and epidemiological data were collected, including age, sex, residence, time of onset, intensity of symptoms, and location of hemorrhagic signs. Patients whose symptoms met the criteria for DF but who had a laboratory-confirmed diagnosis of malaria were excluded.

### Study area, patients and samples

**Figure 1:**
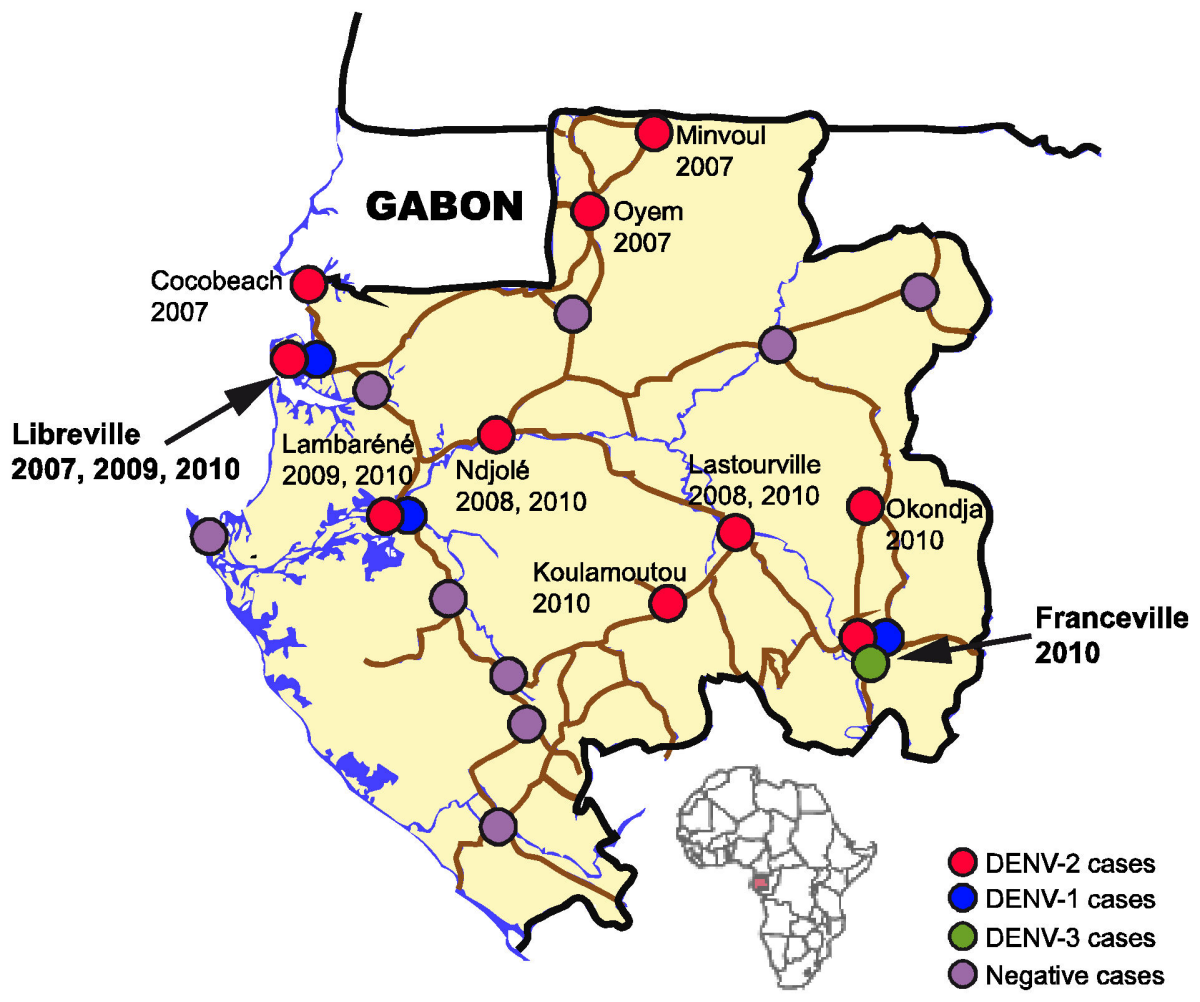
Distribution of DENV types circulating in Gabon between 2007 and 2010. Red circle: DENV-2; Blue circle: DENV-1; Green circle: DENV-3; Purple circle: No DENV; In bold: main towns affected by DENV outbreaks.

Following the 2007 Gabon outbreak [18,19], an active surveillance network for dengue-like syndromes was established throughout the country, between September 2007 and August 2010, in three major sentinel healthcare facilities of the capital city, Libreville, and in the regional hospitals of all major Gabonese towns (Figure 1). One physician in each sentinel healthcare facility acted as the CIRMF correspondent and was in charge of clinical examinations, blood sampling and symptomatic treatment of all suspected clinical cases during the study period.

Blood samples were collected in the healthcare centers in 7-mL Vacutainer tubes containing EDTA (VWR International, Fontenay Sous Bois, France) and were routed to the CIRMF laboratory for further biological diagnosis and investigations. After centrifugation (10 min, 2,000 *g*), plasma was stored in aliquots at -20°C for virological and molecular investigations. Hematological and biochemical tests were performed with a Hematology Analyser ACT 10 (Beckman Coulter, Fulton, CA, USA) and a Hitachi model 902 Automatic Analyser (Roche Diagnostics, Meylan, France). Thick and thin blood films were stained with 20% Giemsa and examined for malaria parasites.

### Molecular and virological investigations

Quantitative real-time PCR (qPCR) targeting the 3’UTR region was performed to detect all four DENV serotypes [21]. A 7500 Real-Time PCR system was used (Applied Biosystems™, Foster City, CA, USA), with a melting temperature of 60°C, 400 nM each primer and 200 nM probe. To validate the qPCR, RNA DENV-positive and negative controls were added from the cDNA synthesis step, using the High Capacity cDNA kit (Applied Biosystems™) according to the manufacturer’s instructions. DENV typing was also performed as previously described [21].

A specific DENV-2 PCR fragment of the envelope (E) gene (758-bp, genome position 1503-2260 nt) was amplified with SuperScript™ III One-Step RT-PCR Systems with Platinum® Taq High Fidelity (Invitrogen, Carlsbard, CA, USA). Specific one-step reverse-transcription PCR (RT-PCR) reactions were run in a final volume of 50 µL with 400 nM primers (Q2R: ARGCVGCYCCRTAGATTG and Q2F: ATGRARTGCTCYCCDAGAAC) in a GeneAmp 9700 Thermal Cycler (Perkin Elmer, Waltham, MA, USA) programmed for 30 min at 45°C, 2 min at 94°C and then 40 cycles of denaturation (30 s, 94°C), primer annealing (30 s, 52°C), primer extension (45 s, 68°C) and a final extension step of 5 min at 68°C.

A consensus DENV-1 and DENV-3 PCR fragment of the envelope (E) gene corresponding to a 472-bp fragment of DENV-1 (genome position 1234-1705 nt) and to a 935-bp fragment of DENV-3 (genome position 1256-2190 nt) was amplified in two steps, as previously described [22].

### Phylogenetic analysis

For phylogenetic analysis, partial 758-bp DENV-2, 468-bp DENV-1 and 935-bp DENV- 3 env (E) sequences were analyzed and then, aligned with dataset combining representative sequences of each DENV types and genotypes available in GenBank. Phylogenetic analyses were performed with MrBayes V.3.2 software, using the default chain for two million generations with the GTR+G+I nucleotide substitution model [23]. Trees were sampled every 10 generations, resulting in 500,000 saved trees, the first 12,500 saved trees being discarded as burn-in. Trees were visualized with FigTree 1.3.1 from the BEAST package available from http://evolve.zoo.ox.ac.uk/beast/, and were also mid-point rooted for clarity. All the Gabonese DENV sequences are shown in bold and are available under GenBank accession numbers JX080294-JX080298, JX080292-JX080293 and JX080299, respectively for DENV-2, DENV-1 and DENV-3 strains.

## Results

### Epidemiological findings

We documented DENV circulation throughout Gabon between 2007 and 2010. In total, 4,287 samples were analyzed and we identified 407 patients infected with DENV, of whom 386 (94.8%) were DENV-2+, 20 (4.9%) were DENV-1+ and one (0.2%) was DENV-3+ (Table 1). The mean age of the patients positive for DENV infections was 32.2 ± 16.2 years (range, 1-85 years), with a F/M sex ratio of 1.3 (data not shown). The mean age of the 386 DENV-2 infected patients was 32.5 ± 16.4 years (range, 1-85 years), with a F/M sex ratio of 1.4. The mean age of the 20 DENV-1 infected patients was 29.2 ± 11.7 years (range, 5-53 years), with a F/M sex ratio of 0.8. The DENV-3 infected patient was a 19-year-old man.

**Table 1:** Dynamic of DENV infections in Gabon between 2007 and 2010.

	**No. DENV-2**	**No. DENV-1**	**No. DENV-3**	**Total**
**Epidemic 2007**	**69**	**1**	**-**	**70**
Libreville	60	1	-	61
Cocobeach	6	-	-	6
Oyem	2	-	-	2
Minvoul	1	-	-	1
**Inter-epidemic 2008**	**11**	**-**	**-**	**11**
Ndjolé	10	-	-	10
Lastourville	1	-	-	1
**Inter-epidemic 2009**	**9**	**13**	**-**	**22**
Lambaréné	9	5	-	14
Libreville	-	8	-	7
**Epidemic 2010**	**297**	**6**	**1**	**304**
Franceville	264	6	1	271
Koulamoutou	21	-	-	21
Libreville	7	-	-	7
Okondja	2	-	-	2
Lambaréné	1	-	-	1
Ndjolé	1	-	-	1
Lastourville	1	-	-	1
**Total (%)**	**386 (94.8)**	**20 (4.9)**	**1 (0.2)**	**407 (100.0)**

DENV: Dengue virus; -: none

### Emergence and spread of DENV

Between 2007 and 2010, DENV infections occurred during the rainy seasons (April to July and November to December; data not shown) and spread from north-west to south-east Gabon (Figure 1). The first epidemic episode in 2007 occurred predominately in the capital city, Libreville (n = 61), and in north-western towns such as Cocobeach (n = 6), Oyem (n = 2) and Minvoul (n = 1) (Table 1, Figure 1). Over the next two years, only sporadic cases were reported, with a north-west to south-east pattern of spread. In 2008, only a few cases were observed, limited to small towns located in the center of the country such as Ndjolé (n = 10) and Lastourville (n = 1). In 2009, sporadic cases occurred in Lambaréné (n = 14) and Libreville (n = 8). In 2010, a second epidemic episode started in Koulamoutou (n = 21) and spread to Franceville (n = 271), a provincial capital in the far south-east of the country. The same year, a few sporadic cases occurred in Libreville (n = 7), Okondja (n = 2), Lambaréné (n = 1), Ndjolé (n = 1) and Lastourville (n = 1).

### Simultaneous circulation of three DENV types

Among the 70 patients diagnosed during the 2007 outbreak centered on Libreville, 69 were DENV-2+ and one was DENV-1+ (Table 1). In 2008, all the 11 patients diagnosed in Ndjolé and Lastourville were DENV-2+. In 2009, among the 22 patients diagnosed in Lambaréné and Libreville, 9 were DENV-2+ and 13 were DENV-1+. Lambaréné was affected by both DENV-2 (n = 9) and DENV-1 (n = 5), while Libreville (n = 8) was only affected by DENV-1. Among the 304 patients diagnosed during the 2010 outbreak centered on Franceville, 297 were DENV-2+ and six were DENV-1+. Finally, a case of DENV-3 infection was detected in Franceville. The same year, the towns of Koulamoutou (n = 21), Libreville (n = 7), Okondja (n = 2), Lambaréné (n = 1), Ndjolé (n = 1) and Lastourville (n = 1) were only affected by DENV-2. No temporal pattern of the three DENV serotypes circulating in Franceville was observed (data not shown).

### Phylogenetic characterization of DENV-2, DENV-1 and DENV-3 strains

**Figure 2:**
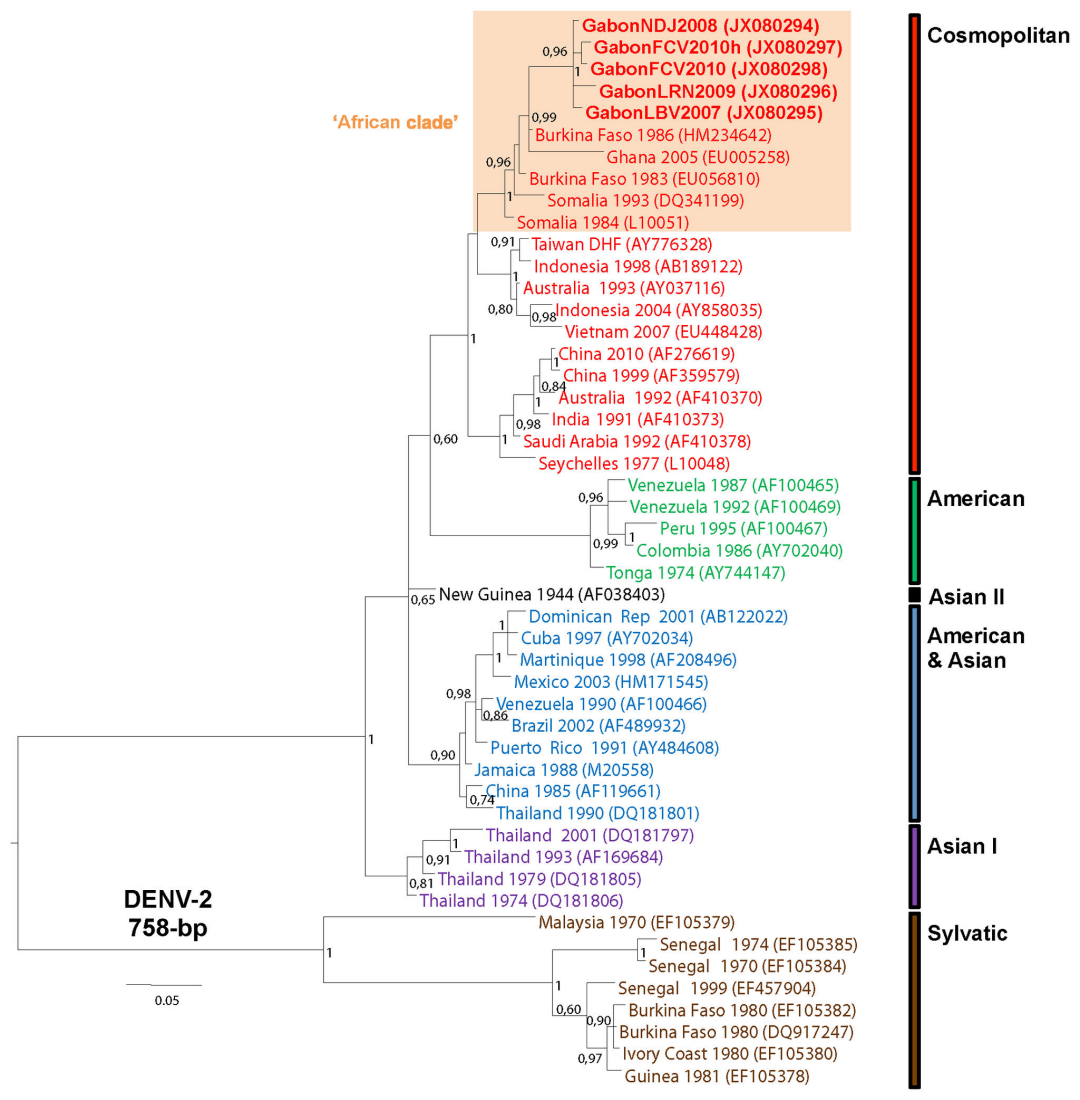
Phylogenic relationships of DENV-2 strains circulating in Gabon between 2007 and 2010. The DENV-2 sequence from patient with hemorrhagic sign ends with ‘h’ (for hemorrhagic sign).

**Figure 3:**
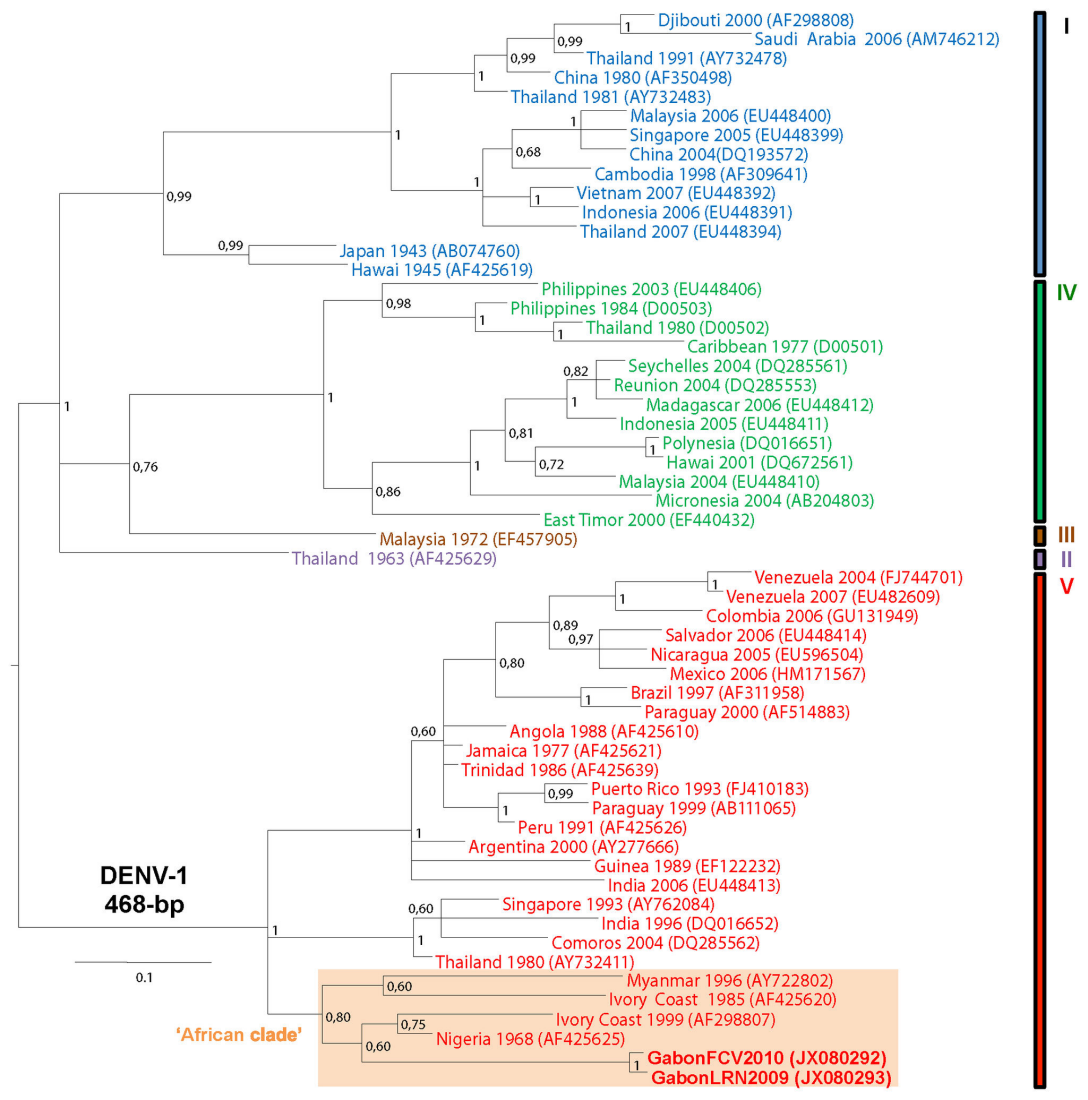
Phylogenic relationships of DENV-1 strains circulating in Gabon between 2009 and 2010.

**Figure 4:**
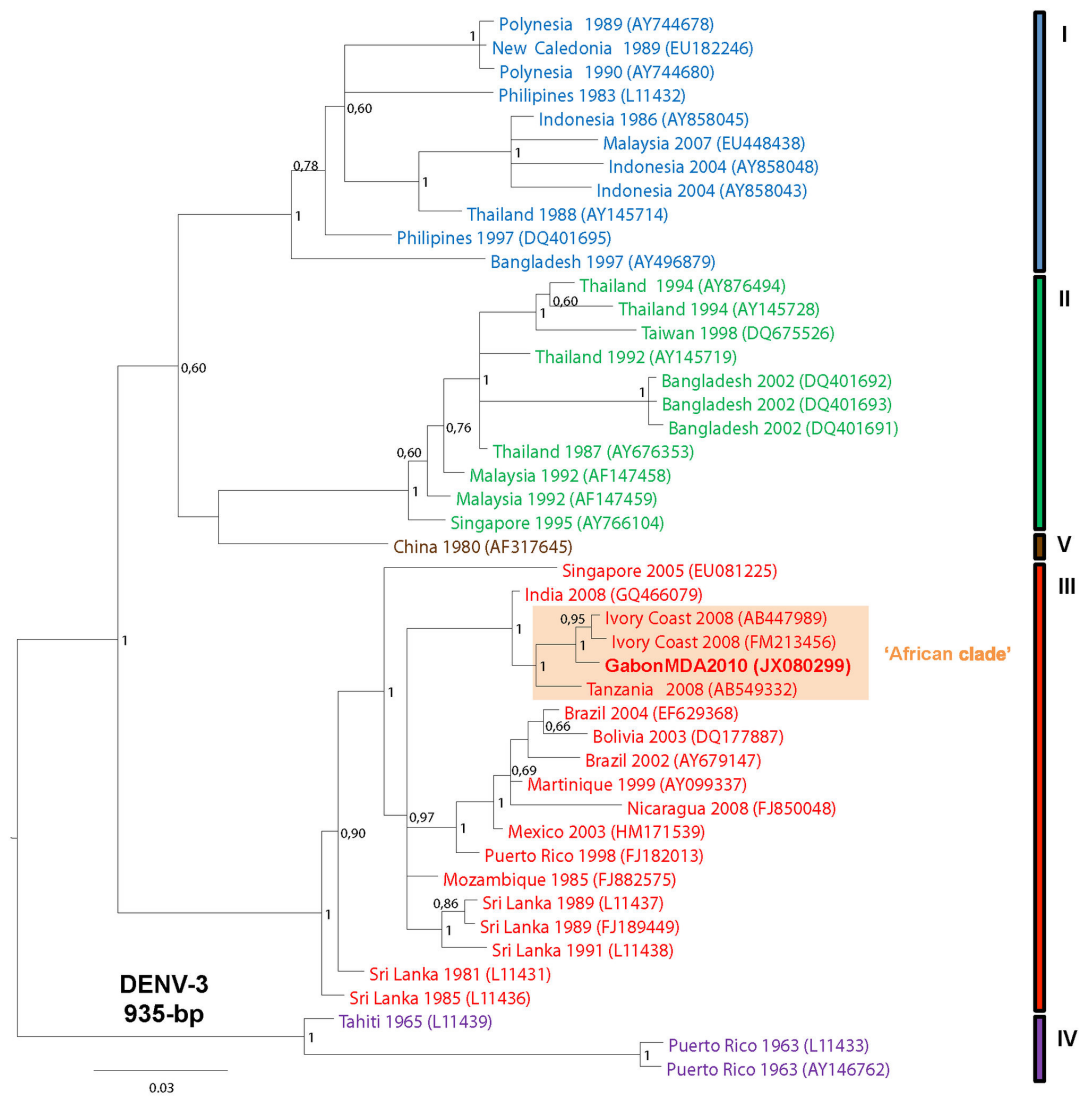
Phylogenic relationships of the DENV-3 strain circulating in Gabon during the 2010 Franceville outbreak.

To characterize the DENV strains circulating between 2007 and 2010 during the epidemic and inter-epidemic episodes, partial envelope (E) gene sequences were phylogenetically compared (Figures 2-4). The Gabonese DENV-2 strains sampled from 2007 to 2010 formed a monophyletic group within the Cosmopolitan genotype (Figure 2). These five strains grouped together with all DENV-2 strains isolated from humans in other parts of Africa, such as Ghana in 2005, Burkina Faso in 1983 and 1986, and Somalia in 1984 and 1993, forming a well supported clade hereafter referred to as the ‘African clade’.

The two Gabonese DENV-1 strains sampled in 2009 and 2010 grouped together and belonged to genotype V. These strains formed a phylogenetic clade with most of the African strains included in this genotype, such as the two Ivorian strains isolated in 1985 and 1999 and the Nigerian strain isolated in 1968 (Figure 3).

The Gabonese DENV-3 strain belonged to genotype III and formed a phylogenetic clade with most of the DENV-3 strains isolated from humans in other parts of Africa, such as the two Ivorian strains in 2008 and the Tanzanian strain in 2010 (Figure 4).

### DENV-2 infections associated with hemorrhagic signs in 2010

Of the 264 laboratory-confirmed cases of DENV-2 infection observed during the 2010 Franceville outbreak, six patients had hemorrhagic signs, with conjunctival injection in three patients, and epistaxis, gingival bleeding and bloody stools in one patient each (Table 2). All the patients were women, aged from 15 to 76 years. All had fever, headache, arthralgia and myalgia, and two had also petechiae. The tourniquet test was negative in all cases, and hemoglobin levels were similar to healthy controls. One of these patients had hyperproteinemia, one had hyperproteinemia and moderate leucopenia, one had both moderate leucopenia and lymphopenia, and one had moderate leucopenia, lymphopenia and thrombocytopenia. None required hospitalization and no deaths were reported (data not shown).

**Table 2:** Clinical characteristics of DF patients with hemorrhagic signs during the 2010 Franceville outbreak.

**Patients (Sex, Age)**	**Symptoms**	**Hemorrhagic signs**	**Tourniquet test**	**Hemoglobin (g/dL)**	**Proteinemia (g/L)**	**Leucocytes (cell/mm^3^)**	**Lymphocytes (cell/mm^3^)**	**Platelets (cell/mm^3^)**
1 (F, 15y)	FHAM	Conjunctival injection	Neg	12.6	ND	3,200*	944*	202,000
2 (F, 21y)	FHAMP	Conjunctival injection	Neg	ND	144*	ND	ND	ND
3 (F, 30y)	FHAM	Conjunctival injection	Neg	12.0	61	6,900	2,553	227,000
4 (F, 44y)	FHAM	Bloody stools	Neg	12.8	60	1,600*	528*	113,000*
5 (F, 53y)	FHAMP	Gingival bleeding	Neg	12.0	97*	4,400	1,804	314,000
6 (F, 76y)	FHAM	Epistaxis	Neg	13.0	104*	3,800*	1,482	239,000

Sex, F: female; Age, y: year; Symptoms, F: fever; H: headache; A: arthralgia; M: myalgia; P: petechiae; Neg: negative; ND: not done; *: outside normal range (Hemoglobin: 12–16 g/dL; Proteinemia: 60–80 g/L; Leucocytes: 4,000–10,000 cell/mm^3^; Lymphocytes: 1,000-4,000 cell/mm^3^; Platelets: 150,000–450,000 cell/mm^3^)

## Discussion

Active surveillance of febrile syndromes in Gabon between 2007 and 2010 showed that DENV-2 spread from north-west to south-east of the country. Following its first detection during the 2007 Gabon outbreak [19], DENV-2 circulated continuously during the rainy seasons, causing clinical cases each year. The rapid spread of DENV-2 throughout the country was probably due to two main factors. First is the extraordinary ability of *Ae. albopictus* to proliferate in multiple habitat types, to supplant endemic species, and to sustain DENV infections in an (sub)urban cycle [24]. Second, there was no significant serological evidence of DENV circulation before 2007 in Gabon [25]. Therefore, the geographic progression of a DENV competent vector within a non-immune population probably enhanced DENV transmissibility. Furthermore, our phylogenetic analyses of partial envelope (E) gene sequences showed a recent common ancestry of the Gabonese DENV-2 strains from 2007 to 2010 and the Gabonese DENV-1 strains from 2009 and 2010. This further suggests that the DF propagation from north-west to south-east Gabon resulted in gradual spread rather than multiple and independent DENV introductions from neighboring endemic countries.

During the three-year study period, we observed a diversification of DENV serotypes in Gabon. Indeed, DENV-1 appeared in 2007 and caused several clinical cases in 2009 and 2010. Then, DENV-3 appeared in 2010 and co-circulated with DENV-2 and DENV-1 during the 2010 Gabon outbreak. To our knowledge, this is the first report of simultaneous circulation of three different DENV serotypes in an African country. Previous DF cases and outbreaks in Africa have mostly been caused by only one of the four DENV serotypes and mainly affected western countries such as Nigeria (1964, DENV-1 & DENV-2), Senegal (1974, 1980, 1986, 1991, 1999, 2008, DENV-2; 1980s, DENV-2 & DENV-4; 2007, 2009, DENV-3), Burkina Faso (1980, 1983, DENV-2; 1985, 2007, DENV-1), Ivory Coast (1980, 2007, DENV-2; 1999, DENV-1; 2008, DENV-3), Guinea (1981, DENV-2), Cameroon (2002, DENV-1; 2006, DENV-3), Gabon (2007, DENV-2), Mali (2008, DENV-2), Cape Verde (2009, DENV-3) and Benin (2010, DENV-3), and eastern countries such as Kenya (1982, DENV-2), Mozambique (1984-85, DENV-3), Soudan (1984-86, DENV-1 & DENV-2), Djibouti (1991-92, DENV-2), Somalia (1992-93, DENV-2 & DENV-3), and Eritrea (2005, Unknown) [26-39].

To date, the African situation contrasts with the Asian and American contexts where DENV is endemic and outbreaks are often due to multiple DENV serotypes [6,8,11]. Nevertheless, the continuous circulation of DENV-2 in Gabon, associated with DENV-1 and DENV-3 serotypes diversification, suggests that DENV is becoming endemic in this country. In addition, our phylogenetic analyses suggest that the Gabonese DENV-2, DENV-1 and DENV-3 strains did not derive directly from Asian strains but rather from strains that had been circulating in other parts of Africa. This indicates genetic evolution within DENV ‘African clades’ in order to adapt to the environment and to local mosquito and mammal species. Despite a few reported outbreaks in Africa, the DENV-2 and DENV-1 ‘African clades’ were probably silently maintained for several decades and have progressively become endemic, while the DENV-3 ‘African clade’ may have been introduced to this continent more recently.

The Gabonese situation might change the DENV observations previously made in Africa such as in areas where DHF/DSS were ultimately described from the introduction of a new DENV strain [40]. In view of the ADE hypothesis [12,13], simultaneous circulation of three different DENV serotypes (DENV-2, DENV-1 and DENV-3) in Gabon could result in severe clinical forms of DF such as DHF/DSS. Interestingly, we observed six cases of DENV-2 infection associated with hemorrhagic signs during the 2010 Gabon outbreak, in which three different DENV serotypes co-circulated. These DENV-2 infections associated with hemorrhagic signs cannot be considered as DHF, because thrombocytopenia was not quite significant and no signs of increased vascular permeability were measured. In front of a public-health emergency, laboratory confirmation for DHF is not consistently available in Africa. In addition, the WHO case definition is widely debated because of evidences of severe clinical forms of DF that have not been characterized as DHF [11,41]. Although these six cases do not meet all standard WHO criteria for DHF, this is the first report of several DF cases associated with hemorrhagic signs during a simultaneous circulation of different DENV serotypes in Africa.

DENV thereby appears to be expanding into new territories in Africa, and will probably become more widely established on this continent. Indeed, it is essential to avoid DHF/DSS in resource-limited settings such as Africa, where all four DENV serotypes have already been detected in human. In the light of disease control and surveillance measures for DF in Asia and the Americas, public-health awareness must be heightened in order to prevent the introduction of new DENV strains, which might lead to a massive DHF/DSS occurrence in Africa.
